# The Effect of Obesity on Repolarization and Other ECG Parameters

**DOI:** 10.3390/jcm13123587

**Published:** 2024-06-19

**Authors:** Irena A. Dykiert, Krzysztof Kraik, Lidia Jurczenko, Paweł Gać, Rafał Poręba, Małgorzata Poręba

**Affiliations:** 1 Department of Physiology and Pathophysiology, Division of Pathophysiology, Wroclaw Medical University, 50-368 Wrocław, Poland; 2 Students’ Scientific Association of Cardiovascular Diseases Prevention, Wroclaw Medical University, 50-368 Wrocław, Poland; 3 Department of Population Health, Division of Environmental Health and Occupational Medicine, Wroclaw Medical University, 50-372 Wrocław, Poland; 4 Department of Internal Medicine, Occupational Diseases, Hypertension and Clinical Oncology, Wroclaw Medical University, 50-556 Wrocław, Poland; 5 Department of Paralympic Sport, Wroclaw University of Health and Sport Sciences, 51-617 Wrocław, Poland

**Keywords:** electrocardiography, repolarization markers, obesity, overweight

## Abstract

**Background**: Overweight and obesity are important risk factors in the development of cardiovascular diseases. New repolarization markers, such as the Tpeak-Tend interval and JTpeak intervals, have not yet been profoundly studied in obese patients. The study aims to analyze whether, in patients with obesity and overweight, repolarization markers, including the Tpeak-Tend interval, are prolonged and simultaneously check the frequency of other ECG pathologies in a 12-lead ECG in this group of patients. **Methods**: A study group consisted of 181 adults (90 females and 91 males) with overweight and first-class obesity. The participants completed a questionnaire, and the ECG was performed and analyzed. **Results**: When analyzing the classic markers, only QT dispersion was significantly higher in obese people. The Tpeak-Tend parameter (97.08 ms ± 23.38 vs. 89.74 ms ± 12.88, respectively), its dispersion, and JTpeak-JTend parameters were statistically significantly longer in the obese group than in the controls. There were also substantial differences in P-wave, QRS duration, and P-wave dispersion, which were the highest in obese people. Tpeak-Tend was positively correlated with body mass and waist circumference, while JTpeak was with BMI, hip circumference, and WHR. Tpeak/JT was positively correlated with WHR and BMI. In backward stepwise multiple regression analysis for JTpeak-WHR, type 2 diabetes and smoking had the highest statistical significance. **Conclusions**: Only selected repolarization markers are significantly prolonged in patients with class 1 obesity and, additionally, in this group, we identified more pathologies of P wave as well as prolonged QRS duration,

## 1. Introduction

Overweight and obesity are characterized by abnormal and excessive adipose tissue deposition in the body [1]. These states are important risk factors in the development of several diseases, including cardiovascular diseases, diabetes, and neoplastic diseases, which are leading causes of death worldwide [2]. They may also increase the risk of death by exacerbating symptoms of respiratory disorders [3] and infectious diseases, including COVID-19 [4,5]. Moreover, overweight and obesity often lead to lowering the quality of life as risk factors for diabetes mellitus, respiratory disorders, and musculoskeletal disease development. In connection with these risks, the governments of numerous countries allocate considerable resources to prevent and treat obesity; for example, the USA’s healthcare system spends about USD 173 billion annually for that purpose only [6].

Nowadays, obesity is considered a separate disease entity, and its prevalence is common; sometimes, the term “a pandemic of obesity” is used [7]. In 2016, globally, about 13% of all adults were classified as obese, and 39% were classified as overweight [1]. The epidemiological situation regarding these states looks very pessimistic. The number of obese people has almost tripled in the last 50 years [7]. In Poland, there is also a continuing trend of increasing incidence of obesity. In 1975, the prevalence of obesity in Polish adults was estimated to be 10.6%, which grew by about 0.3% per year until 2006. After 2006, this trend accelerated to about 0.5% per year. In 2016, the prevalence of obesity in Poland was estimated to be 25.6%. The prevalence of obesity in Polish children and adolescents also become higher over the years, and in 2016, it was estimated to be 9.1%. Similarly, the prevalence of overweight and obesity among children and adolescents increased globally—in 1975, 4.3% were classified as overweight and 0.8% as obese, while in 2016, 18.4% were classified as overweight and 6.8% as obese [8].

A standard 12-lead ECG has numerous clinical applications, including screening for cardiac abnormalities in asymptomatic individuals, diagnosing and monitoring cardiac conditions, assessing response to treatment, guiding medical decisions, and evaluating perioperative risk in surgical patients. In the context of obesity, this condition may be associated with various ECG abnormalities [9,10,11]. ECG findings can provide valuable information for risk stratification, identifying potential complications, and guiding management strategies in obese individuals at risk for cardiovascular disease.

The evidence of the relationship between obesity and ECG findings is inconsistent across studies, showing conflicting results [12,13,14,15]. Until now, most commonly reported ECG findings in obese patients include increased heart rate, prolonged QT interval, increased QRS duration and R wave amplitude, and altered T wave morphology [9,10,11].

In recent years, new repolarization markers have been proposed. The new repolarization markers can be divided into early and late repolarization indices. Early repolarization indices include the JTpeak interval, while late repolarization indices include the Tpeak-Tend interval (Tp-e) and, additionally, the JTpeak/JT, Tp-e/Jtpeak, and Tpeak/JT ratios have been introduced as playing a role in the potential use in patients after myocardial infarction [16]. There are still not many studies establishing the role and clinical significance of the novel repolarization parameters regarding a group of people with obesity and overweight. The Tpeak to Tend interval is one of the most promising novel ventricular repolarization parameters. Tp-e is potentially helpful as a predictor of mortality in patients with heart failure [17,18], as a predictor of cardiac events in long QT syndrome [19,20,21], and its prolongation may be used as a risk factor of ventricular arrhythmia in STEMI patients after percutaneous coronary interventions [22,23]. Current studies are inconsistent regarding whether Tp-e is HR (heart rate)-dependent [24,25]. For this reason, the Tp-e/QT ratio was proposed as an indicator independent of HR.

This study aims to analyze whether, in patients with obesity and overweight, the classic repolarization markers, as well as the novel ones, including the Tpeak-Tend interval, are prolonged. Additionally, we have attempted to determine if other ECG pathologies are present in this group of patients.

## 2. Materials and Methods

### 2.1. Study Population and Trial Design

The study was conducted at the Department of Pathophysiology of Wroclaw Medical University in 2020–2023. The Wroclaw Medical University Ethics Committee approved the study, which was conducted following Good Clinical Practice and the Declaration of Helsinki. The population of the examined patients included adult residents of Wroclaw and its vicinity. We created the initial study group of 303 people out of the adult volunteers who agreed to participate in the study and gave their written consent.

**Figure 1:**
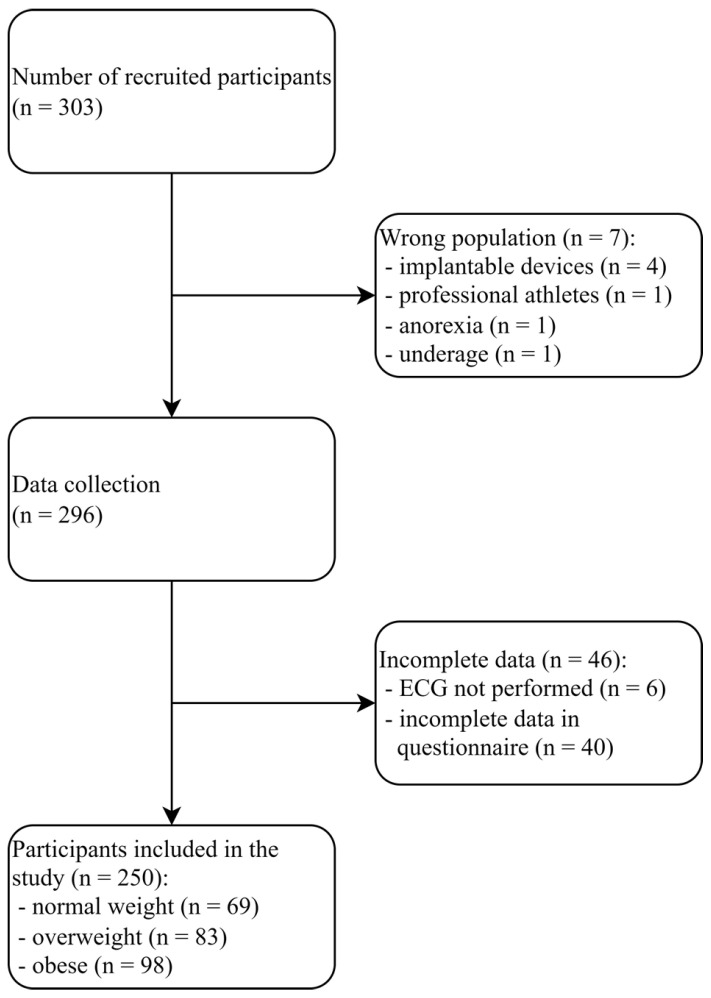
Flowchart presenting the selection of participants.

The inclusion criteria for the study group were age over 18 and BMI above 25. Adult patients with a BMI below or equal to 25 were recruited to the control group. We excluded seven patients due to the following causes: one was underage at the moment of recruitment, one person was an athlete, which could affect the ECG results, and for similar reasons, four patients with implantable devices and one person with a history of anorexia in the questionnaire. After collecting the data, we excluded 46 patients due to incomplete information in the questionnaire or the lack of ECG. Figure 1 presents the process of selection of participants, and the characteristics of the comorbidities are presented in Table 1 and Table 2.

A research group of 181 adults (female/male 90/91) whose BMI exceeds 25 qualified for the study. Among this group, 83 participants were classified as overweight (BMI in the range of 25.0–29.9 kg/m^2^; female/male 41/42), and 98 were classified as obese (BMI equal or higher than 30 kg/m^2^; female/male 49/49). The control group consisted of 69 volunteers (females/males 56/13) with a normal BMI. The mean BMI of the obese patients was 33.6 kg/m^2^, and all participants belonged to the class 1 obesity category; the mean in the group with overweight was 27.5 kg/m^2^, and in the controls, 22.8 kg/m^2^.

The first stage of the research was to fill out a proprietary questionnaire, including questions about physical activity, the use of stimulants, eating habits, comorbidities, and family and psychological history. In the next step, the basic anthropometric measurements were carried out: weight, height, heart rate, and blood pressure. Then, the appropriate calculations were made (including BMI and WHR). Then, a 12-lead ECG was performed. The analysis of electrocardiogram recordings included standard ECG measurements and the novel electrocardiographic markers currently used in literature.

### 2.2. Electrocardiographic Analysis

**Figure 2:**
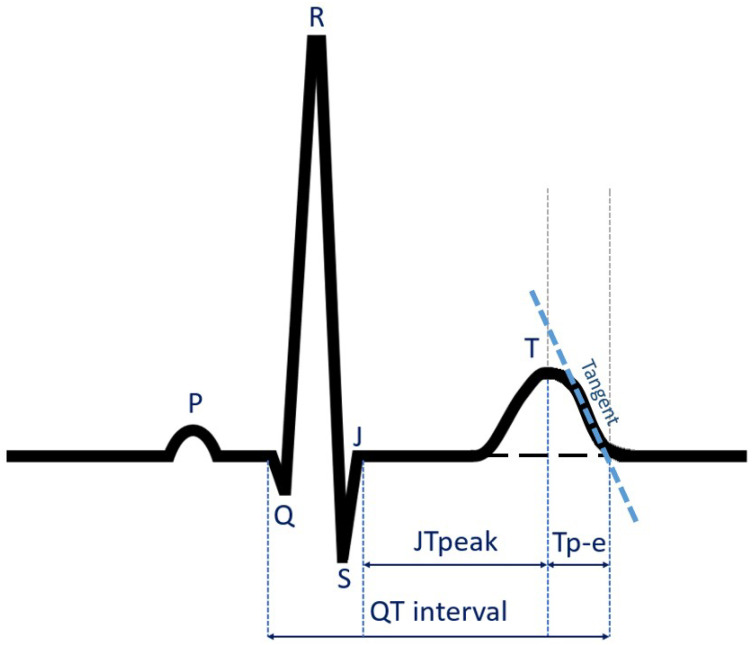
ECG repolarization intervals—QT, JTpeak, Tp-e.

The standard electrocardiographic parameters such as heart rate, P-wave width, P dispersion, PQ interval, QRS complex width, QT interval, QTc interval, and QT dispersion were measured. Novel repolarization parameters measured were: Tpeak-Tend, (Tpeak-Tend) disp, (Tpeak-Tend)/QT, (Tpeak-Tend)/QTc, JTpeak, JT interval, JTpeak/JT, (Tpeak-Tend)/JTpeak, Tpeak, Tpeak/JT, JTpeak-Jtend, and (JTpeak-JTend) dispersion. The Tpeak-Tend was measured using the tangent method based on Rosenthal‘s method [26], as shown in Figure 2. All measured parameters are presented in Table 3.

The electrocardiography was performed using the CardioExpress SL 12 (Spacelabs Health Care Ltd., Hertford, UK) employing the Sentinel cardiology information management system (Spacelabs Health Care 2017 (Sentinel v10.5.0.8939). The 12-lead ECG was performed with a standard chart speed of 25 mm/s and a 10 mm/mV voltage. The acquisition mode of the ECG was 10 s of 12-lead simultaneous recording. The calibration signal input was 1 mV ± 2%, and the sample frequency—1000 Hz. The filters used included an enabled network filter, 0.15 Hz isoline filter, 25 Hz muscle filter, and 100 Hz low-pass filter.

The ECG recordings included between 7 and 21 full ECG cycles, depending on the patient’s heart rate. Two independent researchers, medical students and a physician blinded to the clinical status, performed the ECG measurements. Two qualified cardiologists were in the group of researchers; in any case of problematic ECG recording, the cardiologist finally accepted the results.

### 2.3. Statistical Analysis

The statistical package “Dell Statistica 13.1” (Dell Inc., Round Rock, TX, USA) was used for statistical analysis. The arithmetic means and standard deviations of the estimated parameters were calculated for the quantitative variables. For the 12-lead ECG parameters in the whole study group, the values of the −95.000% confidence interval, +95.000% confidence interval, and coefficients of variability were also calculated. The distribution of variables was examined using the Lilliefors test and the W-Shapiro–Wilk test. The results for qualitative (nominal) variables were expressed as percentages. In comparative analyses, three subgroups of patients were compared: obese, overweight, and normal body mass. Therefore, multiple comparison was used. ANOVA was used for further statistical analysis in the case of quantitative independent variables with a normal distribution. The homogeneity of variances was checked using Levene and Brown–Forsyth tests. In the absence of homogeneity of variances, the Kruskal–Wallis ANOVA test was used to compare the significance of mean differences in 3 subgroups. In the case of variables with a distribution other than normal, the Kruskal–Wallis ANOVA test, a non-parametric equivalent of the analysis of variance, was used for quantitative independent variables. Statistically significant differences between individual arithmetic means were then determined with the Newman–Keuls post hoc test. For independent qualitative variables, multi-way tables and the maximum likelihood chi-square test were used for further statistical analysis. Correlation and regression analyses were performed to determine the relationship between the analyzed variables. In the case of quantitative variables with a normal distribution, Pearson’s r correlation coefficients were determined, and in the case of quantitative variables with a non-normal distribution, Spearman’s r coefficients were determined. The parameters of the models obtained in the backward stepwise multivariable regression analysis were estimated using the least squares method. The results were statistically significant at *p* < 0.05.

## 3. Results

### 3.1. Baseline Characteristic

The mean age in the entire study group was 59.94 ± 13.22, with a BMI of 28.64 ± 4.99. In the study group, 104 patients (41.6%) were men, and 146 were female (58.4%). The mean BMI was 28.64 ± 4.99. The comorbidities in the study group are shown in Table 1. When divided into subgroups, obesity A vs. overweight B and control group C, the mean BMI for the subgroups were 33.62, 27.56, and 22.86, respectively. There were no statistically significant differences in WHR. However, significant differences were noted in waist values: 107.29, 95.29, and 79.72, respectively, and in hip circumference: 115.23, 104.26, and 93.83, respectively. In the subgroups with obesity and overweight, hypertension was significantly more commonly present even in 64.3% of patients with obesity as well as type 2 diabetes, and the highest incidence was in patients with obesity, ranging to 21.4%.

Table 1 and Table 2 summarize the study group and subgroups’ baseline characteristics. On analyzing the regular medication use in the whole study group, it was found that 16.4% (41 persons) of participants were on thyroid hormones, 27% (68 persons) were on beta-blockers, 14.5 (36 persons) were on dihydropyridine calcium channel blockers, and 36.4% (91 persons) declared the use of other drugs. Among them, there were patients after stroke and myocardial infarction who declared acetylsalicylic acid, patients with paroxysmal atrial fibrillation 8% (22 patients) were on NOAC treatment, 13.2% (33 patients) were on oral medication for diabetes treatment, mainly biguanides; few patients declared other drugs such ACE inhibitors and proton-pump inhibitors.

### 3.2. Analysis of 12-Lead ECG Parameters in Studied Subgroups

Statistically significant differences were found in subsequent subgroups in P-wave width, with the highest values for obesity and overweight groups (A 113.12 ± 19.98 ms, B 111.66 ± 17.92 ms) as well as in the case of P-wave dispersion, which was the highest for obese people (A 40.08 ± 19.39 ms, B 31.01 ± 21.58 ms, 30.59 ± 18.66 ms). There were also differences in the PQ interval, which was the longest for the obese people but still within the norm (A 177.45 ± 29.74 ms, B 167.73 ± 28.92 ms, C 155.58 ± 29.86 ms). QRS complex width was the highest for obese people and statistically longer than in controls (A 107.24 ± 21.34 ms, B 102.47 ± 23.26 ms, C 100.14 ± 13.42 ms, p A vs. C).

When analyzing the classic depolarization and repolarization markers, slight differences in QT and QTc intervals were observed. However, they were not significant, and only QT dispersion was significantly higher in obese people when compared to patients with overweight and normal body mass (A 39.63 ± 23.14 ms, 32.02 ± 27.95 ms, C 32.06 ± 20.77 ms). All data are presented in Table 4.

Taking into account the novel electrocardiographic parameters, we found that in the whole study group, the mean Tpeak to Tend interval was 94.66 ± 21.28 ms, (Tpeak-Tend) dispersion was 39.17 ± 19.38 ms, (Tpeak-Tend)/QT was 0.24 ± 0.05 ms and (Tpeak-Tend)/QTc was 0.22 ± 0.04 ms. All the novel parameters of repolarization are presented in Table 3, together with classical parameters. Confidence intervals and coefficients of variability of ECG parameters in the entire study group were also presented in Table 3. Tpeak-Tend and its dispersion were statistically significantly longer in the obese group than in the control group. Additionally, the JTpeak-JTend parameter was significantly longer in obese patients than in people with normal body mass.

The differences in repolarization markers are shown in Table 4.

### 3.3. Linear Relationship between Body Mass Parameters and 12-Lead ECG Parameters in the Entire Study Group

There were positive linear correlations between both atrial parameters, P-wave and PQ interval, and some body mass parameters, that is, body mass, BMI, waist and hip circumference, and between P dispersion and BMI, waist and hip circumferences. Moreover, a relationship existed between QRS complex width and body weight, BMI, and waist circumference.

Amongst novel electrocardiographic parameters, Tpeak-Tend was positively correlated with body mass and waist circumference, while JTpeak was associated with BMI, hip circumference, and WHR. Additionally, Tpeak was correlated with WHR. Also, Tpeak/JT was positively correlated with WHR and BMI. The correlations are summarized in Table 5.

### 3.4. Backward Stepwise Multiple Regression Model

After implementing a backward stepwise multivariable regression model for JTpeak and Tpeak/JT as dependent variables, we assessed the specific models presented in Table 6 and Table 7.

For the 12-lead ECG JTpeak, age, WHR, type 2 diabetes, and smoking had the highest statistical significance (*p* for the model *p* < 0.001), as for the Tpeak/JT as the dependent variable, male sex and BMI had a positive effect on the model. Β-blockers had a negative impact on the model (*p* for the model *p* < 0.001).

The backward stepwise multivariable regression model is summarized in Table 6 and Table 7.

**Figure 3:**
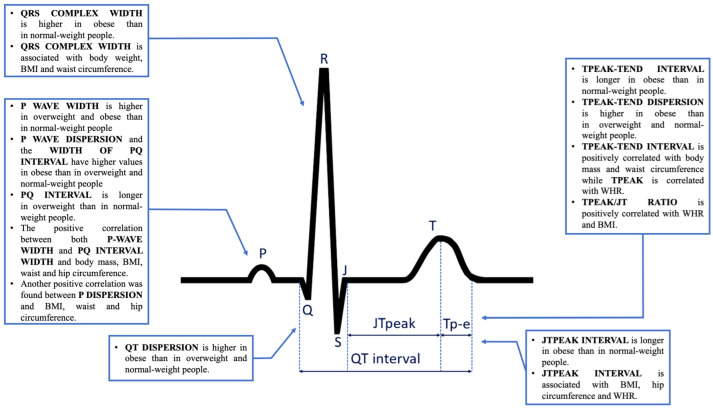
Effects of obesity and overweight on repolarization and other ECG parameters in our study.

The summary of the results of our research and the effects of obesity and overweight on repolarization and other ECG parameters are presented in Figure 3.

## 4. Discussion

The current study investigated the alterations of ECG parameters in people with overweight and obesity, especially the ones concerning repolarization parameters. We found an increase in P-wave dispersion and QRS complex width in obese individuals and an increase in P-wave width and PQ interval in both overweight and obese individuals. Moreover, taking into consideration classic repolarization parameters, we found that obese individuals have significantly higher values of QT dispersion, and analyzing novel repolarization parameters, we found that Tp-e interval, Tp-e dispersion, and JTpeak-JTend have substantially higher values in obese individuals. Our study found that alterations in repolarization parameters in obese individuals are marked mostly in novel rather than classic repolarization parameters. This may indicate the potential clinical use of parameters such as Tp-e interval, Tp-e dispersion, and JTpeak-JTend after standardizing the normal values of these parameters. We also found positive linear correlations between ECG parameters (P-wave width, PQ interval, QRS complex width, Tp-e interval, JTpeak, Tpeak amplitude) and body mass parameters (body mass, BMI, waist circumference, hip circumference, WHR), which may be related to electrophysiological changes present in obese people secondary to remodeling of the myocardium in both atria and ventricles.

Other studies investigating ECG changes in obese people also reported an increased prevalence of left ventricular hypertrophy, left atrial enlargement, and left axis deviation in obese patients, which may indicate structural changes in the heart [9,10]. These findings suggest potential alterations in cardiac electrophysiology, myocardial function, and ventricular repolarization in obese individuals. We did not observe the criteria for left ventricular hypertrophy, which is more common in obese people, even though hypertension was the most common in this group. However, the study group should be highlighted as being comprised mainly of class 1 obesity patients. The most significant electrocardiographic changes could be expected in patients from classes 2 and 3, where chamber overload and enlargements are higher, more comorbidities are identified, and cardiovascular risk is higher.

Regarding classic repolarization parameters, Omran et al.’s meta analysis found that obesity or overweight is related to an increase in the length of QT and QTc intervals and QTc dispersion. Moreover, weight loss was able to revert these alterations [11]. Seyfeli et al. also associated increased QTc dispersion with obesity in women [27]. In Kumar et al.’s study, obese adults aged 18–40 had significantly higher width of QT intervals than adults without obesity. Moreover, Kumar et al. associated prolongation of QT interval with a higher risk of left ventricular hypertrophy and ventricular fibrillation [10].

Furthermore, Waheed et al. found that obese people have wider QTc interval than normal-weight people and associated this prolongation with increased cardiovascular and all-cause mortality [28]. Our study showed slight differences in QT and QTc intervals between BMI groups. However, the trend of these changes was consistent with the conclusions of the previously mentioned studies. On the contrary, Braschi et al. found no significant differences in classical repolarization parameters between normal-weight people and people with uncomplicated overweight or obesity. Moreover, they found a trend in QT dispersion that increased with BMI without reaching the significance condition [14]. Our study also found this trend, and it was statistically significant. Furthermore, Guo et al. associated the prolongation of QTc with metabolic syndrome, which often co-exists with obesity [29]. Analyzing the abovementioned studies to estimate the potential risk, we should consider the class of obesity and co-existing comorbidities, which increase with the increase in body mass.

There are still not many research studies investigating the changes in novel repolarization parameters in overweight and obese individuals. The results of previous studies on these parameters are not consistent. Inanir et al.’s study found that the novel repolarization parameters Tp-e interval, Tp-e/QT, Tp-e/QTc, Tp-e/JT, and Tp-e/JTc are significantly higher in individuals with BMI ≥ 40 than in individuals with normal body weight [12]. Moreover, Bağcı et al. found that the alterations concerning the Tp-e interval and Tp-e/QT and Tp-e/QTc ratios progress gradually with the growth of BMI [13]. Our study partly supports these results regarding Tp-e interval width. However, we found no significant differences between BMI groups regarding Tp-e/QT, Tp-e/QTc, and Tp-e/JT ratios. Contrary to these studies and ours, a study conducted by Al-Mosawi et al. found that Tp-e interval width decreased with the growth of BMI, although this change did not reach significance [15]. Al-Mosawi et al.’s study is the only study we have found with this negative relationship between Tp-e width and BMI. Furthermore, Braschi et al.’s study found no significant changes in Tp-e interval, Tp-e dispersion, and Tp-e/QT ratio between groups of normal-weight people, people with uncomplicated overweight, and people with uncomplicated obesity [14].

Kosar et al. found that P-wave dispersion was increased in obese individuals and that P-wave dispersion was correlated positively with BMI. They also found that obese people had higher values of maximal P-wave duration than normal-weight people. They hypothesized that obesity might be a factor leading to the development of atrial fibrillation [30]. Moreover, Cosgun et al.’s study associated obesity with no other comorbidities to the increase in maximal values of P-wave width and the prolongation of P-wave dispersion [31]. Bocchi et al.’s study also associated BMI and abdominal obesity with an increase in P-wave dispersion [32], and Seyfeli et al.’s study associated obesity with an increase in P-wave dispersion [27]. Our research also found that P-wave duration and dispersion have higher values in overweight and obese people. Therefore, our study supports previously mentioned studies.

Furthermore, Russo et al. found that P-wave dispersion can be significantly reduced by bariatric surgery in morbidly obese patients without comorbidities [33]. Similarly, weight loss due to diet and medical therapy or diet only resulted in decreased P-wave duration and dispersion [34,35]. Prolonging P-wave width or dispersion is associated with a higher risk of developing supraventricular arrhythmias, including atrial fibrillation [36,37,38,39,40,41,42,43,44].

Another alteration reported in obese people’s ECG is a prolongation of QRS complex in comparison to normal-weight people [45,46]. Furthermore, a recent study by Sobhani et al. also found that higher BMI was associated with prolonged QRS complex [47]. Our study supports these findings. We also observed that obese people have a statistically significant increase in QRS complex duration compared with people with normal weight.

Additionally, children and adolescents with abdominal obesity were revealed to have longer PQ intervals, wider QRS complex, and leftward shifts in frontal P-wave, QRS, and T-wave axes in comparison to normal-weight children adolescents. In this group, a positive correlation between PQ interval and QRS duration and BMI, waist circumference, and WHR was also found [48]. We found similar changes in the adult population: an increase in PQ interval and QRS complex width in obese and similar positive correlations between ECG and body weight parameters.

Apart from the ECG parameters examined in this study, there are others that are potentially useful in practice, which we did not take under study. Among them is a microvolt T-wave alternans, potentially useful for patients with coronary artery disease [49]. However, this method has several limitations. Applying this method requires special equipment and the proper heart rate.

There are multiple theories explaining the changes in ECG repolarization parameters due to obesity. Firstly, the changes in P-wave and T-wave morphology may be associated with myocardial fibrosis of ventricles or within the atria [50]. Secondly, obesity may affect ion channels, which may change the potential of myocytes [51]. Obesity may influence I_Na_, I_Ca,L_, and I_to_ ion channels, increasing the risk of long QT syndrome and atrial fibrillation in obese patients. According to Aromolaran et al., the candidates for modulation by obesity are cardiac, such as the abovementioned ion channels and Ca handling proteins. However, the underlying mechanisms of such interactions remain incompletely understood [51]. In research studies on the relationships between obesity and atrial fibrillation in mice, it has been found that the process was partly mediated by a combined effect of sodium, potassium, and calcium channel remodeling and atrial fibrosis [52].

Moreover, mitochondrial antioxidant therapy reduced atrial fibrillation burden, restoring I_Na_, I_Ca,L_, and I_Kur_, resulting in shorter action potential duration and reversed atrial fibrosis. Obesity may be connected with fibrosis and the increased secretion of pro-inflammatory cytokines, hyperglycemia, and insulin resistance, leading to electrical remodeling and thus predisposing to arrhythmias [51]. Additionally, the adipose tissue is associated with subcutaneous and visceral fat accumulation, causing distinct signaling mechanisms. Eventually, some differences may be present in the regional distribution of fat deposits, affecting ion channel/Ca handling protein expression. Other authors found that cardiomyocytes of obese and diabetic patients have increased lipid accumulation, which contributes to the pathophysiology of heart failure and arrhythmia [53,54]. It is known that diabetes quite commonly co-exists with obesity; even in our study subgroup with class 1 obesity, it was identified in 21% of patients. Morrow et al. demonstrated on transgenic models that cardiac lipid overload causes spontaneous arrhythmias, and Purohit et al. revealed that oxidative stress may partly mediate the arrhythmogenic effect [55,56]. Furthermore, in other studies, authors have found that cardiomyocyte lipid overload may increase oxidative stress by activating the protein NOX2, causing mitochondrial dysfunction and abnormalities of internal calcium handling, promoting arrhythmia [57]. More experimental studies in this area are needed.

Obesity may affect survival, and it has been proven in numerous studies that it is associated with the increased risk of several diseases and death, particularly from cardiovascular diseases and cancer; however, only grade 2 and 3 obesity was associated with significantly higher all-cause mortality [58,59,60]. Interestingly, in a comprehensive meta-analysis, it was shown that patients with low weight and overweight had a higher mortality risk during acute coronary syndrome than normal-weight patients [61]. The results showed the U-shaped nonlinear association detected between body mass index and mortality risk with higher mortality risk for BMI < 21.5 kg/m^2^ and >40 kg/m^2^. In contrast, the lowest mortality risk was detected at approximately 30 kg/m^2^, called the “obesity paradox” effect. Additionally, it has been clearly shown that the most severe clinical complications and increase in risk are dedicated to class 3 obesity, which is also called high-risk obesity. In such patients, we may expect the most frequent remodeling of the heart muscle and, secondarily, ECG changes and arrhythmias. From this point of view, class 1 obesity and overweight are theoretically connected with not-severe initial stages changes within the cardiovascular system and heart muscle, resulting in less frequent and minor ECG pathologies.

The association between obesity and cardiovascular diseases has been widely studied. However, this issue is still not fully understood and is complex. Discussing briefly several methods determining cardiovascular risk in obese people, several data present the risk of obese patients in the context of coronary artery disease. Even metabolically healthy obese subjects have a higher incidence of subclinical coronary artery atherosclerosis when compared to normal-weight individuals, which was diagnosed by the calcium scores in cardiac computer tomography (CCT). Furthermore, every 1 kg/m^2^ increase in BMI led to a 5–7% increase in the incidence of CAD across all BMI categories [62,63]. CCT has relatively good sensitivity and specificity; however, even using modern and up-to-date equipment could not always guarantee high image quality for overweight or obese patients [64]. Echocardiography also needs a good visualization, which may be impaired in this group of patients. In uncomplicated obesity cases, the enlarged left ventricular mass in echocardiography might often be an early adaptation of cardiac function, compensating for the greater hemodynamic and metabolic demand. It should be underlined that increased body mass leads to increased metabolic requirements, which may be a step towards the development of CAD [65]. Single-photon emission CT (SPECT) is used in lower-weight patients and avoided in patients whose BMI is more than 35 kg/m^2^ [66]. However, in some studies in which obese people were participating, it was found that, although the obese had a higher risk profile than their non-obese counterparts, obesity was not an independent predictor of abnormal MPS (myocardial perfusion SPECT), raising the possibility that other risk factors associated with obesity (e.g., diabetes) have a much higher impact on the occurrence of coronary artery disease than obesity per se [67]. Nevertheless, there are some limitations of this technique in the obese. Electrocardiography is extensively available and cheap, so it is the first-line test. The common ECG changes in obese people have been commented on within this article, mainly including the increased heart rate, which has not been proven in our study, as we only noted insignificant differences. Other typical pathologies include increased QRS and QT interval. In light of CAD, there are no specific parameters in obese people that could be proposed as specific prognostic markers, especially for obese people. It is noteworthy that the baseline ECG may be influenced by obesity, especially in more advanced obesity stages. ST-T changes are found due to ventricular hypertrophy and overload, which may perplex the diagnostic process [68]. For this reason, non-invasive testing for CAD often has a suboptimal performance.

It is also worth mentioning that being overweight or obese is not the only factor that impacts changes in repolarization parameters. Other factors include the effects of the autonomic nervous system, hormonal metabolism, especially steroid hormones and sex hormones, hyper- and hypokalemia, other electrolyte disorders, using medications, medical procedures performed, and metabolic diseases [69,70]. Moreover, the influence of genetic factors is also possible, e.g., by modifying the operation of ion channels, as in congenital long QT syndrome (LQTS). There are also reports of the potential impact of hyperventilation on disturbances in ventricular repolarization [71]. The influence of air pollution, especially PM_2.5_, cytokines, stress and emotions, and the menstrual cycle’s influence on ventricular repolarization cannot be definitively denied [69,72,73,74].

A growing number of drugs are influencing ventricular repolarization and prolonging the QT interval, potentially also new electrocardiographic repolarization markers. In this group, there are numerous medications, including noncardiac ones. In our study group, patients did not declare any anti-arrhythmic drugs having a significant impact on repolarization; however, some minor relations could have happened, which may have been a confounding factor to some extent. As presented in Table 2, 64%, 50%, and 39% of patients had hypertension, consecutively in obesity, overweight, and normal-weight subgroups. However, only a few participants were treated with ACE inhibitors, which may have a beneficial and protective effect on repolarization and affect the results. Sixty-eight patients (27.2%) were treated with beta-blockers, which also have beneficial activity.

Moreover, we found a negative relation in regression analysis between beta-blockers and one of the repolarization markers (Tpeak/JT) [75,76]. It may explain slight differences in some repolarization parameters between the studied subgroups. It is also possible that some other agents used by patients could affect the repolarization. The majority of agents may have a potential influence on repolarization; one example may be varenicline, approved to help in smoking cessation, which led to prolongation of ventricular repolarization parameters QTc, Tp-e, and Tp-e/QTc ratio [77]. However, in our study group, no one declared the use of this drug. Additionally, 16% of our study group also proclaimed the use of thyroid hormones and 14% of calcium channel blockers, mainly nifedipine and lercanidipine, which may have some effect. More and more evidence is gathered on the relationship between various medications and repolarization markers; however, when patients use various drugs and agents in real-life clinical conditions, the ultimate effect may be complex and unpredictable.

The significance of our study assumes that it may increase our knowledge of pathophysiological changes in the cardiovascular system, especially within the heart and its electrical system function in people with obesity and overweight, as there are still some controversies. Mainly, we have found more pathologies connected to repolarization in patients with class 2 obesity, and probably further studies should employ more patients with class 3 obesity, in whom we expect more cardiovascular and non-cardiovascular complications. The study may contribute to improving the understanding of the role of repolarization indices with the increase in body weight even in the setting of the usual physician’s practice, as the analysis of the electrocardiogram is frequently rather superficial. Numerous studies, including this one, are focused on the detailed ECG examination. It is possible that in the future, Tp-e and its derivates will also be included in computed electrocardiogram analysis, and all the markers, including the classic and the novel ones, will be presented in the report.

### 4.1. Limitations

Our study also has some limitations. We analyzed the 12-lead ECG only once for every participant. Therefore, we could not observe changes in ECG in the long term. Furthermore, the Polish population is mostly ethnically monogenic and does not include minorities. Therefore, we cannot guarantee that the results of our study are universal for all populations. The other limitation mentioned in the last paragraph of the Discussion addresses the medications used by some of the study participants, mainly antihypertensive ones. Potentially, it may constitute a confounding factor.

### 4.2. Future Perspectives

Despite emerging trends and relationships, current research still has many inconsistencies regarding novel repolarization parameters. There is a need for studies with large-scale research and control groups. Thanks to large-scale studies, it would be possible to distinguish subgroups based on age and smaller ranges of BMI (e.g., distinction of alterations in every obesity class). Especially within the study group, there should be more patients with class 2 and 3 obesity in the future perspective.

Furthermore, examining these relationships in more homogenous subgroups, such as diabetes mellitus and hypertension, would also allow for a better understanding of studied ECG alterations. Eventually, ECG, as a simple and easily feasible, as well as widely available, technique, may serve as a first-line tool to estimate the initial pathologies and indicate the increasing cardiovascular risk in obese patients. Paying attention to even minor changes could help to select patients at higher risk.

## 5. Conclusions

We can hypothesize that considering all the limitations and confounding factors, the results we have analyzed may be addressed to class 1 obesity and overweight people.

In patients with class 1 obesity, only QT dispersion was significantly higher in obese people when compared to patients with overweight and normal body mass, and QTc was only insignificantly higher.

The novel repolarization indices, Tpeak-Tend, and its dispersion were statistically significantly longer in the obese group than in the control group, and the JTpeak-JTend parameter was considerably longer in obese patients. Additionally, Tpeak-Tend was positively correlated with body mass and waist circumference.

We revealed significant differences in P-wave and QRS duration and P-wave dispersion in obese people with class 1 obesity, with positive correlations between these parameters and anthropometric parameters such as BMI and waist and hip circumferences.

This study is the introduction for further research on novel electrocardiographic parameters in the future, that is, the Tpeak-Tend and its derivates, and especially interesting would be employing more patients with class 3 obesity, where the number of cardiovascular and non-cardiovascular complications increases.

## Figures and Tables

**Table 1:** Clinical characteristics of the entire study group.

Parameter	%/n or Mean ± SD
age (years)	59.94 ± 13.22
sex (%/n)	
Male	41.6/104
Female	58.4/146
height (cm)	167.37 ± 9.76
weight (kg)	80.42 ± 17.50
BMI (kg/m^2^)	28.64 ± 4.99
waist circumference (cm)	95.99 ± 14.26
hip circumference (cm)	106.60 ± 12.16
WHR	0.95 ± 0.74
hypertension (%/n)	52.8/132
myocardial infarction (%/n)	6.4/16
stroke (%/n)	2.8/7
atrial fibrillation (%/n)	8.8/22
deep vein thrombosis (%/n)	3.6/9
type 2 diabetes (%/n)	13.2/33
thyroid disease (%/n)	16.4/41
smoking (%/n)	13.2/33

BMI—body mass index, WHR—waist-hip ratio.

**Table 2:** Clinical characteristics of the studied subgroups.

Parameter	Obesity (A, n = 98)	Overweight (B, n = 83)	Control Group (C, n = 69)	*p* < 0.05
age (years)	61.18 ± 11.07	53.40 ± 13.70	58.83 ± 15.33	ns
sex (%/n)				
Male	50.0/49	50.6/42	18.8/13	A, B vs. C
Female	50.0/49	49.4/41	81.2/56	A, B vs. C
height (cm)	168.14 ± 9.65	168.64 ± 10.36	164.76 ± 8.78	ns
weight (kg)	95.09 ± 13.24	78.39 ± 11.10	62.02 ± 7.99	A vs. B, C B vs. C
BMI (kg/m^2^)	33.62 ± 3.26	27.56 ± 1.34	22.86 ± 1.71	A vs. B, C B vs. C
waist circumference (cm)	107.29 ± 10.16	95.29 ± 8.55	79.72 ± 7.55	A vs. B, C B vs. C
hip circumference (cm)	115.23 ± 7.32	104.26 ± 5.23	93.83 ± 14.14	A vs. B, C B vs. C
WHR	0.93 ± 0.08	0.91 ± 0.09	1.05 ± 1.56	ns
hypertension (%/n)	64.3/63	50.6/42	39.1/27	A, B vs. C
myocardial infarction (%/n)	7.1/7	7.2/6	4.3/3	ns
stroke (%/n)	2.0/2	3.6/3	2.9/2	ns
atrial fibrillation (%/n)	8.2/8	12.0/10	5.8/4	ns
deep vein thrombosis (%/n)	7.1/7	1.2/1	1.4/1	ns
type 2 diabetes (%/n)	21.4/21	10.8/9	4.3/3	A vs. C
thyroid disease (%/n)	16.3/16	13.2/11	20.3/14	ns
smoking (%/n)	9.2/9	15.8/13	15.9/11	ns

BMI—body mass index, WHR—waist-hip ratio, ns—not significant.

**Table 3:** 12-lead ECG parameters in the entire study group.

Parameter	Mean	Confidence Interval −95.000%	Confidence Interval +95.000%	SD	Coefficients of Variability
HR (bpm)	66.73	66.25	68.21	11.87	17.79
P-wave width (ms)	109.63	107.18	112.08	19.65	17.92
P disp (ms)	34.45	31.91	36.99	20.38	59.16
PQ interval (ms)	168.19	164.37	172.01	30.68	18.24
QRS complex width (ms)	103.70	101.17	106.23	20.34	19.62
QT interval (ms)	389.79	385.72	393.86	32.69	8.39
QTc interval (ms)	408.54	405.37	411.71	25.46	6.23
QTd (ms)	35.02	31.97	38.06	24.45	69.82
QRS axis (°)	26.24	21.09	31.38	41.31	157.46
Sokolow–Lyon index LV (mm)	18.40	17.67	19.13	5.87	31.93
Sokolow–Lyon index RV (mm)	3.63	3.27	3.99	2.26	62.20
Tpeak-Tend (ms)	94.66	92.01	97.31	21.28	22.48
(Tpeak-Tend) disp (ms)	39.17	36.76	41.59	19.38	49.46
(Tpeak-Tend)/QT	0.24	0.23	0.24	0.05	23.28
(Tpeak-Tend)/QTc	0.22	0.22	0.23	0.04	19.35
JTpeak (ms)	199.89	195.92	203.85	31.83	15.92
JT interval (ms)	293.54	289.39	297.70	33.34	11.36
JTpeak/JT	0.69	0.68	0.69	0.07	10.03
(Tpeak-Tend)/JTpeak	0.42	0.40	0.44	0.17	40.93
Tpeak (mV)	0.40	0.37	0.43	0.24	61.05
Tpeak/JT (mV/ms)	0.00	0.00	0.00	0.00	62.92
JTpeak-JTend (ms)	95.93	92.37	99.49	28.57	29.79
(JTpeak-JTend) disp (ms)	44.90	41.23	48.56	29.34	65.36

HR—heart rate, P disp—P wave dispersion, QTc interval—corrected QT interval, QTd—QTd interval dispersion, Sokolow–Lyon index LV—Sokolow–Lyon criteria for left ventricular hypertrophy, Sokolow–Lyon index RV—Sokolow–Lyon criteria for right ventricular hypertrophy, (Tpeak-Tend) disp—Tpeak-Tend dispersion, (JTpeak-JTend) disp—Jtpeak-JTend dispersion.

**Table 4:** Parameters of the 12-lead ECG recording in the studied subgroups.

Parameter	Obesity (A, n = 98)	Overweight (B, n = 83)	Control Group (C, n = 69)	*p* < 0.05
HR (bpm)	66.50 ± 11.45	66.04 ± 12.10	67.90 ± 12.26	ns
P-wave width (ms)	113.12 ± 19.98	111.66 ± 17.92	102.22 ± 19.45	A, B vs. C
P disp (ms)	40.08 ± 19.39	31.01 ± 21.58	30.59 ± 18.66	A vs. B, C
PQ interval (ms)	177.45 ± 29.74	167.73 ± 28.92	155.58 ± 29.86	A vs. B, C B vs. C
QRS complex width (ms)	107.24 ± 21.34	102.47 ± 23.26	100.14 ± 13.42	A vs. C
QT interval (ms)	392.66 ± 25.77	390.19 ± 40.85	385.23 ± 30.32	ns
QTc interval (ms)	411.50 ± 23.43	406.95 ± 30.23	406.25 ± 21.61	ns
QTd (ms)	39.63 ± 23.14	32.02 ± 27.95	32.06 ± 20.77	A vs. B, C
QRS axis (°)	17.32 ± 38.36	27.05 ± 41.37	37.93 ± 42.80	ns
Sokolow-index LV (mm)	17.11 ± 5.15	19.77 ± 6.16	18.58 ± 6.15	ns
Sokolow-index RV (mm)	3.73 ± 2.23	3.32 ± 2.40	3.71 ± 2.22	ns
Tpeak-Tend (ms)	97.08 ± 23.38	95.88 ± 23.71	89.74 ± 12.88	A vs. C
(Tpeak-Tend) disp (ms)	43.29 ± 24.14	37.34 ±17.75	35.52 ± 11.03	A vs. B, C
(Tpeak-Tend)/QT	0.23 ± 0.05	0.25 ± 0.07	0.23 ± 0.03	ns
(Tpeak-Tend)/QTc	0.22 ± 0.04	0.23 ± 0.05	0.22 ± 0.03	ns
JTpeak (ms)	205.92 ± 28.04	198.77 ± 32.39	192.67 ± 34.88	A vs. C
JT interval (ms)	292.82 ± 28.67	295.52 ± 36.14	292.20 ± 36.25	ns
JTpeak/JT	0.69 ± 0.07	0.67 ± 0.07	0.69 ± 0.05	ns
(Tpeak-Tend)/JTpeak	0.46 ± 0.16	0.35 ± 0.21	0.45 ± 0.11	ns
Tpeak (mV)	0.39 ± 0.26	0.40 ± 0.25	0.41 ± 0.21	ns
Tpeak/JT (mV/ms)	0.00 ± 0.00	0.00 ± 0.00	0.00 ± 0.00	ns
JTpeak-JTend (ms)	99.55 ± 34.53	95.98 ± 29.64	90.72 ± 13.54	A vs. C
(JTpeak-JTend) disp (ms)	48.22 ± 37.60	44.04 ± 26.32	41.19 ± 16.33	ns

HR—heart rate, P disp—P wave dispersion, QTc interval—corrected QT interval, QTd—QTd interval dispersion, Sokolow–Lyon index LV—Sokolow–Lyon criteria for left ventricular hypertrophy, Sokolow–Lyon index RV—Sokolow–Lyon criteria for right ventricular hypertrophy, (Tpeak-Tend) disp—Tpeak-Tend dispersion, (JTpeak-JTend) disp—Jtpeak-JTend dispersion; ns—not significant.

**Table 5:** Linear relationships between body weight parameters and 12-lead ECG parameters in the entire study group.

Parameter	Body Weight (kg)	BMI (kg/m^2^)	Waist Circumference (cm)	Hip Circumference (cm)	WHR
HR (bpm)	ns	ns	ns	ns	ns
P-wave width (ms)	0.31	0.25	0.30	0.20	ns
P disp (ms)	ns	0.15	0.16	0.17	ns
PQ interval (ms)	0.38	0.33	0.40	0.32	ns
QRS complex width (ms)	0.16	0.16	0.14	ns	ns
QT interval (ms)	ns	ns	ns	ns	ns
QTTc interval (ms)	ns	ns	ns	ns	ns
QTd (ms)	ns	ns	ns	ns	ns
QRS axis (°)	ns	ns	ns	ns	ns
Sokolow-index LV (mm)	ns	ns	ns	ns	ns
Sokolow-index RV (mm)	ns	ns	ns	ns	ns
Tpeak-Tend (ms)	0.16	ns	0.16	ns	ns
(Tpeak-Tend) disp (ms)	ns	ns	ns	ns	ns
(Tpeak-Tend)/QT	ns	ns	ns	ns	ns
(Tpeak-Tend)/QTc	ns	ns	ns	ns	ns
JTpeak (ms)	ns	0.15	ns	0.19	0.18
JT interval (ms)	ns	ns	ns	ns	ns
JTpeak/JT	ns	ns	ns	ns	ns
(Tpeak-Tend)/JTpeak	ns	ns	ns	ns	ns
Tpeak (mV)	ns	ns	ns	ns	0.16
Tpeak/JT (mV/ms)	ns	0.15	ns	ns	0.16
JTpeak-JTend (ms)	ns	ns	ns	ns	ns
(JTpeak-JTend) disp (ms)	ns	ns	ns	ns	ns

HR—heart rate, P disp—P wave dispersion, QTc interval—corrected QT interval, QTd—QTd interval dispersion, Sokolow–Lyon index LV—Sokolow–Lyon criteria for left ventricular hypertrophy, Sokolow–Lyon index RV—Sokolow–Lyon criteria for right ventricular hypertrophy, (Tpeak-Tend) disp—Tpeak-Tend dispersion, (JTpeak-JTend) disp—Jtpeak-JTend dispersion, ns—not significant.

**Table 6:** Backward stepwise multivariable regression model in the entire study group for JTpeak (ms) as the dependent variable.

	Age	WHR	Type 2 Diabetes	Smoking
Regression coefficient (RC)	0.439	17.563	13.064	6.259
SEM of Rc	0.163	3.032	6.081	2.803
*p*	<0.01	<0.001	<0.05	<0.05
*p* for the model	*p* < 0.001			

**Table 7:** Backward stepwise multivariable regression model in the entire study group for Tpeak/JT (mV/ms) as the dependent variable.

	Male	BMI (kg/m^2^)	β-Blockers
Regression coefficient (RC)	0.001	0.001	−0.001
SEM of Rc	0.000	0.000	0.000
*p*	<0.001	<0.001	<0.05
*p* for the model	*p* < 0.001		

## Data Availability

The data are not publicly available due to patients’ privacy.
